# Microbial Signatures in The Rodent Eyes With Retinal Dysfunction and Diabetic Retinopathy

**DOI:** 10.1167/iovs.63.1.5

**Published:** 2022-01-05

**Authors:** Ram Prasad, Bright Asare-Bediko, Angela Harbour, Jason L Floyd, Dibyendu Chakraborty, Yaqian Duan, Regina Lamendella, Justin Wright, Maria B. Grant

**Affiliations:** 1 Department of Ophthalmology and Visual Sciences, University of Alabama at Birmingham, Birmingham, Alabama, United States; 2 Department of Anatomy, Cell Biology & Physiology, Indiana University School of Medicine, Indianapolis, Indiana, United States; 3 Department of Endocrinology, The Second Affiliated Hospital of Chongqing Medical University, Chongqing, China; 4 Wright Labs, LLC, Huntingdon, Pennsylvania, United States

**Keywords:** ocular microbiome, gut, diabetic retinopathy, type 1 diabetes

## Abstract

**Purpose:**

The gut microbiome has been linked to disease pathogenesis through their interaction in metabolic, endocrine, and immune functions. The goal of this study was to determine whether the gut and plasma microbiota could transfer microbes to the retina in type 1 diabetic mice with retinopathy.

**Methods:**

We analyzed the fecal, plasma, whole globe, and retina microbiome in Akita mice and compared with age-matched wild-type (WT) mice using 16S rRNA sequencing and metatranscriptomic analysis. To eliminate the contribution of the ocular surface and plasma microbiome, mice were perfused with sterile saline solution, the whole globes were extracted, and the neural retina was removed under sterile conditions for retinal microbiome.

**Results:**

Our microbiome analysis revealed that Akita mice demonstrated a distinct pattern of microbes within each source: feces, plasma, whole globes, and retina. WT mice and Akita mice experienced transient bacteremia in the plasma and retina. Bacteria were identified in the retina of the Akita mice, specifically *Corynebacterium, Pseudomonas, Lactobacillus*, *Staphylococcus*, *Enterococcus*, and *Bacillus.* Significantly increased levels of peptidoglycan (0.036 ± 0.001 vs. 0.023 ± 0.002; *P* < 0.002) and TLR2 (3.47 ± 0.15 vs. 1.99 ± 0.07; *P* < 0.0001) were observed in the retina of Akita mice compared to WT. Increased IBA^+^ cells in the retina, reduced a- and b-waves on electroretinography, and increased acellular capillary formation demonstrated the presence of retinopathy in the Akita cohort compared to WT mice.

**Conclusions:**

Together, our findings suggest that transient bacteremia exists in the plasma and retina of both cohorts. The bacteria found in Akita mice are distinct from WT mice and may contribute to development of retinal inflammation and barrier dysfunction in retinopathy.

Since 2007, after the establishment of the human microbiome project, wide knowledge of microbes and their roles in the maintenance of homeostatic events including their contribution in disease progression has accumulated.^1^ Trillions of bacteria have made the human body their permanent residence, entangled with the human genome itself, and are considered as the last human organ under active research.^2^ In 1938, Philip B. Price defined bacteria and categorized them as resident or transient bacteria.^3^ Microorganisms that occupy the organ of the body are called the resident bacteria, whereas those coming from distant organs were named as transient bacteria. Initially the concept of resident or transient bacteria was introduced in the context of skin, but later, with the advancement of technology and multiomics, knowledge of the microbial composition and their functional roles grew in other organs.

The human intestine is the largest ecosystem of the microbiome in the body.^4^ Gut microbes not only contribute to physiological and metabolic processes but also contribute to the development of the immune system.^4^^,^^5^ Studies have shown that changes in the composition of the gut microbiome, through either the increase or decrease of specific bacterial diversity and richness, are linked to the pathogenesis and progression of many diseases including obesity, cancer, autoimmunity, diabetes, cardiovascular disease, and atherosclerosis.^6^^–^^8^ In autoimmune disease and inflammatory conditions, such as type 1 diabetes (T1D), certain bacterial populations infect the gut epithelial barrier layer resulting in its shedding and impact its function by generation of exotoxins.^9^ Disruption to the gut epithelial and endothelial barriers leads to increased intestinal permeability and translocation of gut microbial peptides, food antigens and intestinal toxins into the bloodstream.^10^ Recently, we demonstrated a distinct pattern of the gut microbial population in feces of Akita (T1D mouse model)^11^ and *db/db* (T2D mouse model) mice^12^ compared with their age-matched control littermates. We showed an alteration in alpha and beta diversity in Akita mice and an enrichment in the *Bifidobacterium* spp. that generate peptidoglycan (PGN). PGN levels were found threefold higher in the blood compared to wild-type (WT) mice.^11^

Diabetic retinopathy (DR), a leading cause of irreversible blindness, is considered a disease of the microvascular and neural retina and affects approximately 90% of T1D and 50% to 60% percent of T2D subjects.^13^^,^^14^ In addition to its detrimental effects on vision, the presence of DR is often the harbinger of widespread systemic vascular complications, with cardiovascular disease being the leading cause of death among diabetic patients.^15^ Furthermore, the impact and relevance of DR is rising as the prevalence of diabetes increases with both increasing life expectancy of diabetic patients and increased incidence of the diagnosis. Between 2010 and 2050 the number of Americans with DR is expected to be double and reach over 14 million diabetics. Diabetes has now been deemed a global health crisis by the World Health Organization (National Eye Institute). Chronic systemic inflammation is the key mechanistic pathway of DR and creates an environment within the retina that leads to vascular endothelial compromise and increased permeability. Other factors that contribute to DR include chronic hyperglycemia, end glycosylation products, hypertension, and oxidative stress.

The impact of altered gut microbiome on retinal inflammation is incompletely understood. Our previous studies in diabetic animal models led us to ask whether the retina was similarly impacted by transient bacteremia as other tissues. Gut microbes have been identified in human plasma^16^; thus we postulated that gut microbes and microbial peptides, translocated into the systemic circulation via an impaired gut barrier and could enter the retina via a disrupted blood retinal barrier in DR. Therefore, in the current study, we characterize the gut and plasma microbiome and asked whether microbes from the plasma could enter the retina of Akita and WT mice. We characterized the plasma and retina microbes in both cohorts using 16S rRNA sequencing and metatranscriptomic analysis. We also determined the levels of the microbial peptide, PGN, in the plasma and retina in both experimental cohorts.

## Materials and Methods

### Experimental Animals

Akita mice (*Ins2*^WT/C96Y^) (n = 11) and their WT (n = 11) littermates were generated in the animal facility at University of Alabama at Birmingham (UAB) using an in-house breeding scheme. Akita mice develop diabetes at about five or six weeks of age.^17^ Experimental mice from both cohorts were maintained under standard housing conditions of 12-hour dark/12-hour light cycle, room temperature of 24° ± 2° C, and 50% ± 10% humidity. Food and water were provided to the animals as desired. Experimental protocol for the animal study was approved by the Institutional Animal Care and Use Committee at the University of Alabama (APN-21196). Metabolic characteristics and genotyping of the WT and Akita mice have been previously published.^11^

### Samples Collection for Microbiome Analysis

#### Feces, Plasma and Whole Globes

Feces and plasma from Akita (n = 3) and WT (n = 3) cohorts were collected under the sterile condition in the class II laboratory hood. Briefly, mice were held in hand and squeezed gently to collect the feces samples directly into nuclease free tubes and snap frozen in liquid nitrogen and stored at −80°C. All specimens were collected by study personnel in a standardized fashion using sterile gloves. Blood was withdrawn through cardiac puncture using sterile needle and syringe and collected in commercially available sterile anticoagulant tubes. Plasma from each sample was obtained after centrifugation at 1500*g* for 10 minutes and collected in nuclease-free tubes.

The whole globe was enucleated under sterile conditions in the class II laboratory hood. A dry calcium alginate swab was passed four times along the upper, lower palpebral, caruncle, and fornix conjunctiva using a disposable aseptic dry cotton swab.^18^ Another aseptic dry cotton swab containing the topical anesthetic was used as a blank control. Each swab was then placed into nuclease free sterile cryogenic tubes (Thermo Scientific) and snap frozen in liquid nitrogen and store at −80°C. All samples were shipped in dry ice to Wright Labs, LLC. for 16S rRNA sequencing (V3-V4 region). Bacterial DNA was isolated as described by Duan et al.^11^

### Retina Isolation for 16S rRNA Analysis

Akita (n = 8) and WT (n = 8) mice were either perfused with 0.9% sterile saline solution to eliminate the plasma microbiome (n = 4) or not perfused (n = 4). To avoid any possible contamination including environmental and surface ocular microbiome, eyeballs were enucleated under the sterile conditions in a class II cell culture hood. Briefly after enucleation, globes were cleaned by topical application of anti-bacterial solution (betadine; 10%) for three minutes, extensively washed in 70% ethanol and flooded with sterile saline. Sterile cotton swabs were then used to swab the surface of the globe to test for 16S rRNA to determine that the cleansing procedure resulted in removal of the ocular surface microbiome. This cleansing procedure was performed so that when the retinas were removed any accidental touching of the external surface of the eye would not result in contamination of the retina with ocular surface bacteria. Each swab was then placed into nuclease free sterile cryogenic tubes (Thermo Fisher Scientific, Waltham, MA, USA) and snap frozen in liquid nitrogen and stored at −80°C. The carefully cleaned globes were then dissected using autoclaved instruments and the anterior chamber and lens removed. Each retina (n = 4) was removed using sterile forceps and immediately transferred into nuclease free vials and snap frozen in liquid nitrogen and stored until used for 16S rRNA analysis. The cotton swabs, used to wipe the surface of the globes were also sequenced for 16S rRNA and serve as a control. Sterile swabs that did not touch any surface were used a negative controls.

### 16S rRNA Gene Sequencing and Microbiome Analysis

Approximately 0.25 µg genomic DNAs per sample were extracted using DNA Isolation kit following the manufacturer's instructions (MoBio, Carlsbad, CA, USA). The number of total bacteria was quantified using quantitative real time polymerase chain reaction (qRT-PCR) as described previously.^11^ After quantification using the Qubit High Sensitivity dsDNA kit (Life Technologies, Carlsbad, CA, USA) and dilution, pooled libraries were loaded on an Illumina MiSeq V2 500 cycle kit cassette with 16S rRNA library sequencing primers and set for 250 base pair, paired end reads. Raw sequence data was successfully obtained; paired-end sequences were trimmed at a length of 250 bp, and quality control was set as an expected error of less than 0.005 by USEARCH V7.

After sequencing, the obtained reads were analyzed by the QIIME 1.9.0 software (http://qiime.org/).^19^ The USEARCH61 algorithm was used to identify chimeric sequences and to pick open reference operational taxonomic units. Taxonomy was assigned using the Greengenes 16S rRNA gene database (13-5 release, 97%) and organized into a BIOM formatted OUT table, which was summarized within QIIME 1.9.0. Singletons and doubletons were removed from the dataset before the initiation of any diversity analysis.

### Alpha and Beta Diversity Analysis

The plots of alpha and beta diversity were generated by GraphPad Prism, R-studio software, and QIIME-1.9.0 sequence analysis package. Rarefactions were performed in all samples using a minimum depth 460 and a maximum depth of 4600 and a step size 460. Alpha diversity values were calculated for the Observed Species, Heip's Evenness, and Chao 1 Index metrics. Significance between genotypes were assessed using nonparametric t-tests, with 999 Monte Carlo permutations.

Beta diversity analyses were conducted after the ASV table had first undergone cumulative sum scaling normalization^20^ to mitigate differences between samples based on sequencing depth. Distances between samples were calculated using the Weighted Unifrac metric^21^ based on the normalized table and rooted tree. The resulting distance matrix was visualized as a partial least squares-discriminant analysis (PLS-DA) and plotted using R-studio software. PLS-DA was conducted for the 16S rRNA dataset in all four sample fractions (fecal, plasma, eyes, and perfused retina).

### Metatranscriptomic Analysis

Metatranscriptomic analysis was conducted in Akita and WT mice as described by Duan et al.^11^ The transcriptome library was prepared followed by quality check using a high-sensitivity bioanalyzer chip (Agilent Technology, Tokyo, Japan). MetaPhlAn, a metatranscriptomic analysis tool, was implemented to quantify the taxonomic profile in each sample. To obtain functional gene expression, data was annotated using the Uniref90 database within Humann2. Uniref90 annotations were regrouped as KEGG Orthology (KO) terms, which consequently underwent counts per million normalization within Humann2 for stratified bar plot analysis and LefSe enrichment plots.

### Quantification of Peptidoglycan in the Retina

The levels of PGN in the tissue homogenates of the retina samples were measured using a colorimetric mouse peptidoglycan (no. MBS263268; MyBioSource Inc., San Diego, CA, USA) assay kit following the manufacturer's protocol. The absorbance was measured at 450 nm using a microplate reader, and the levels of PGN were calculated as per the standard curve. To avoid the volumetric differences, total protein was measured in the tissue homogenate prepared from retina of Akita and WT mice, and PGN levels were calculated and expressed as ng/mg protein.

### Measurement of Ocular TLR-2 Levels

The levels of TLR-2 were determined by ELISA using retina homogenates (no. ab224880; Abcam, Cambridge, MA, USA) following the manufacturer's protocol. The absorbance was measured at 450nm using a microplate reader, and the levels of TLR-2 were calculated as per the standard curve and expressed as pg/mg protein.

### Immunostaining of Iba-1^+^ Cells and VCAM-1 Expression in Retina

Immunohistochemical staining of mouse retinas was performed according to a previously published protocol.^22^ Briefly after euthanization of mice, eyes were enucleated and fixed in 4% paraformaldehyde (PFA) for 15 min. After fixation, eyes were washed in PBS, cornea and lenses were removed and mount in OCT medium and immediately frozen on dry ice. Frozen blocks were sectioned at 5 µm thickness using cryostat.

Frozen sections were thawed at room temperature one hour, washed in PBS, and permeabilized with 0.25% Triton-X in PBS for 5 min at room temperature. Sections were blocked with 5% horse serum for 2 h then incubated with primary antibodies specific for Iba-1 and vascular cell adhesion molecule 1 (VCAM-1) overnight at 4° C. Sections were then washed and incubated in fluorescent-labeled secondary antibodies for one hour at room temperature, followed by washing for five minutes at room temperature. Sections were than mounted with anti-fade mounting medium and imaged using a Zeiss microscope (Zeiss, Oberkochen, Germany) equipped with ZEN software. Intensity of Iba-1^+^ cells and VCAM-1 expression were quantified by ImageJ software. Image analysis was completed in a masked fashion.

### Electroretinography

To determine retinal function, electroretinography (ERG) was performed on Akita and WT mice after overnight dark adaption using LKC Bigshot ERG system.^22^ Before starting the ERG, mice were anesthetized with ketamine (80 mg/kg body weight) and xylazine (15 mg/kg body weight). Eyes were dilated with atropine/phenylephrine under dim red light. Scotopic rod signaling was assessed with 10 increasing intensities of white light. Photopic cone signaling was assessed with four increasing light intensities. Responses were averaged and analyzed using the LKC EM software.

### Acellular Capillaries Quantification

Eyes were fixed in 4% formalin and trypsin digest were prepared for analysis of acellular capillaries as previously published.^22^ Briefly, eyes were enucleated and incubated in 4% paraformaldehyde overnight. Retinas were isolated, washed and digested in elastase solution (40 Units elastase/mL; Sigma-Aldrich, St. Louis, MO, USA) to remove the non-vascular tissue. The vascular beds were mounted on glass slides followed by staining with periodic acid–Schiff's base and hematoxylin. Five to six fields from the central to mid-periphery were imaged, and the number of acellular capillaries per square millimeter were quantified.

### Statistical Analysis

Data were evaluated for the presence of outliers and adherence to a normal distribution using GraphPad Prism, version 8.1 software. Statistical significance of normally and non-normally distributed data were assessed via Student's *t* test at *P* = 0.05. The nonparametric Kruskal–Wallis and pairwise Wilcoxon tests were used for microbiota LefSe enrichment analysis. The data sets were considered significantly different if the *P* value was <0.05. Multiple permutations (1000) were conducted for ANOSIM and PERMANOVA analysis of beta diversity clustering between cohorts, as well as all alpha diversity pairwise comparisons.

## Results

### Microbial Abundance in Target Tissues

**Figure 1.**
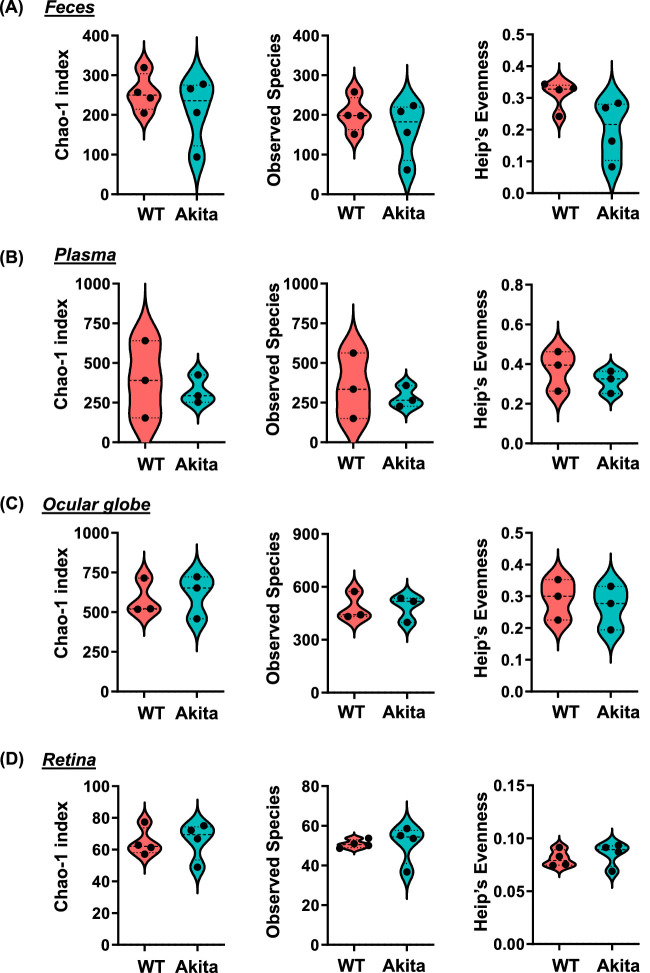
Alpha diversity in Akita mice. Alpha diversity violin plots compared the Chao-1 index, observed species, and Heip's evenness between feces **(A)**, plasma **(B)**, globes **(C)**, and retina **(D)** from Akita and WT mice. Each *dot* represents individual sample.

We determine the alpha and beta diversity in feces, plasma, and whole globes (Figs. 1A–1C). To assess 16S rRNA in the retina, Akita and WT mice littermates were either perfused with saline (n = 4 each cohort) or not perfused (n = 4 each cohort) and then their retina extracted under sterile conditions (Fig 1D). Alpha diversity represent differences in microbial richness whereas beta diversity is the heterogeneity among the samples. We observed changes in alpha diversity using observed species, chao1 index, and Heip's evenness metrics. These metrics represent nonparametric methods to determine the number of species in the community. As seen in Figure 1, we did not observe any significant difference in the alpha diversity of the fecal, plasma, globes and retina of the Akita mice compared with WT mice.

**Figure 2.**
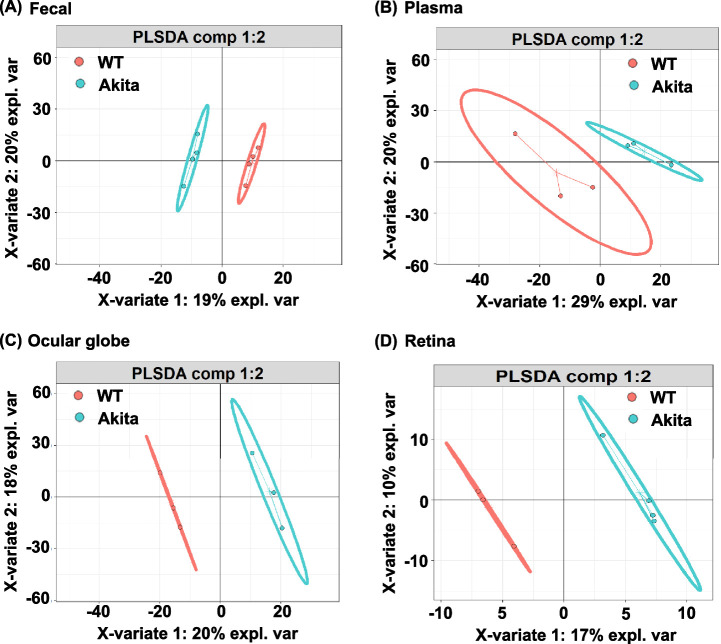
Beta diversity in Akita mice. Beta diversity PLS-DA plots showing phylogenetic community compositions within each genotype between feces **(A)**, plasma **(B)**, globes **(C)**, and retina **(D)** from Akita and WT mice. Each *dot* represents individual sample.

Beta-diversity quantifies dissimilarity in community composition between samples or intra-individual divergence. As seen in Figure 2, two different clustering were observed in feces, plasma, globes, and retina between Akita and WT mice. In the Akita mice, beta diversity was reduced in feces compared WT mice (Fig. 2A). The PLS-DA plots shows enrichment in plasma, globes, and retina of Akita mice compared to their WT counterparts (Figs. 2B–2D). These observations suggest that increased beta diversity in plasma, eyes, and retina could be the outcome of impaired gut barriers and dysbiosis that facilitate the translation of gut microbes and microbial peptide into systemic circulation.^11^

### Phylogenic Diversity and Composition

**Figure 3.**
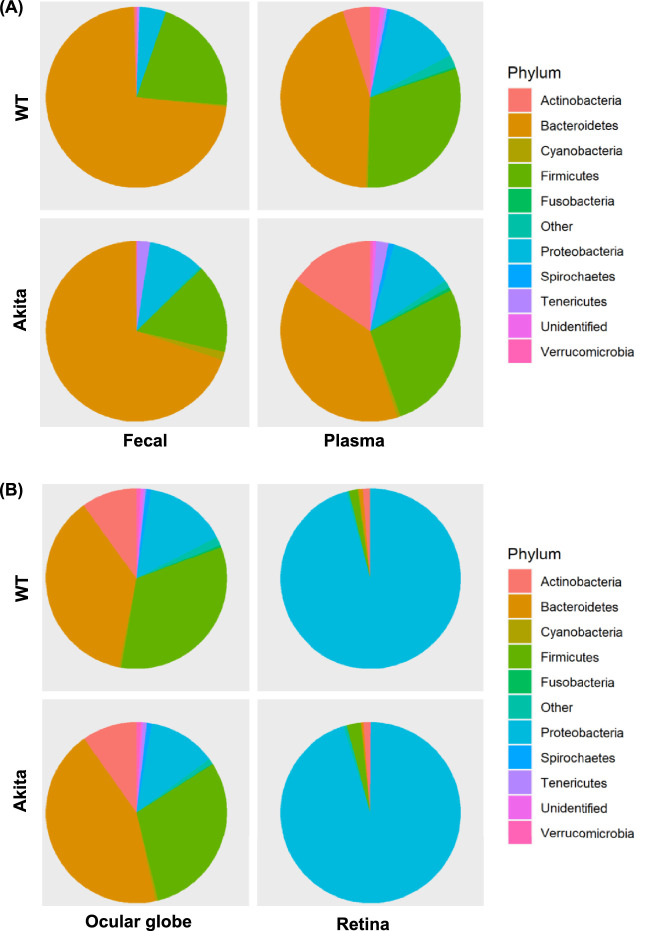
Phylogenic variation and composition in feces, plasma, globes, and retina from Akita and WT mice. Counts per million–normalized counts of MetaPhlAn displayed differential abundances of prominent taxa **(A)**. The ratio of Firmicutes and Bacteroidetes was calculated in feces and plasma, of Akita mice and compared with WT mice **(B)**. Phylogenic differences in globes and retina of Akita and WT mice. For preparation of ocular globes, the 16S rRNA of the ocular surface microbiome was subtracted from analysis of the globe. For the preparation of the retinas, Mice were perfused with sterile saline solution, the ocular surface microbiome was remove using chemical and mechanical washes and then the retinas were dissected and removed under sterile conditions.

Next, microbial communities were clustered based on their phylogenic differences. The relative abundances of *Actinobacteria, Bacteroidetes, Cyanobacteria, Firmicutes, Fusobacteria, Proteobacteria, Spirochaetes, Tenericutes*, and *Verrucomicrobia* in the feces and plasma of Akita and WT mice are shown in Figure 3A. *Actinobacteria, Bacteroidetes, Firmicutes,* and *Proteobacteria* were the dominant phylum of the globes (Fig. 3B) and plasma samples (Fig. 3A), and in the feces *Actinobacteria* was almost undetectable*.* The higher abundance of *Bacteroidetes* and lower abundance of *Firmicutes, Fusobacteria,* and *Proteobacteria* were observed in the globes of Akita mice compared to the WT cohort (Fig. 3A).

Bacteria belonging to *Firmicutes* and *Bacteroidetes* phylum are the most dominant (approximately 90%) in the gut and influence function. Increased Firmicutes/Bacteroidetes (F/B) ratio has been observed in obesity, whereas decreased F/B ratio contributes to selected inflammatory disorders.^23^ We observed a reduced F/B ratio in the globes (71.53 ± 11.23 vs. 92.88 ± 23.63) and feces (25.81 ± 11.88 vs. 30.59 ± 8.11) of Akita mice, while increased ratio in plasma of Akita mice (84.56 ± 27.6 vs. 71.96 ± 10.2) compared to WT mice (Fig. 3B).

A lower abundance of *Bacteroidetes* was observed in the retina of Akita mice compared to WT mice, while a higher abundance of *Firmicutes* and *Verrucomicrobia* was seen*. Verrucomicrobia* was not detected in WT mice*.* The *Actinobacteria and Proteobacteria* remain unchanged (Fig. 3B). An increased F/B ratio was observed in retina of Akita mice compared to their WT littermates.

### Identification of Dominant Genera in Fecal, Plasma, Globes and Retina of Diabetic Mice

**Figure 4.**
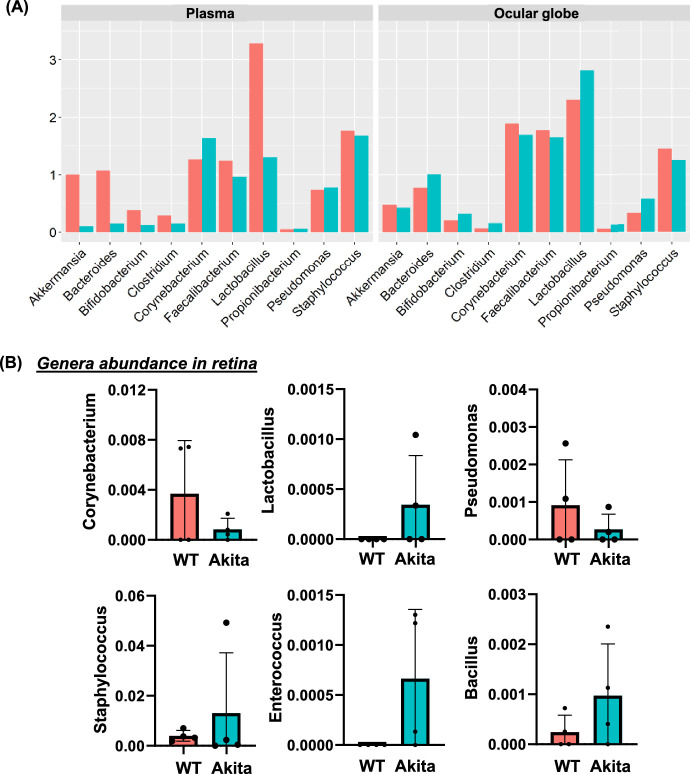
Identification of bacterial species in the feces, plasma, globes and retina in Akita mice. The 16S rRNA sequencing identified top 10 genera in plasma and globes of both cohorts **(A)**. Only six genera were detected in retina of Akita and WT mice **(B)**.

Under ideal conditions, each organ has its own specific microbiome, considered the resident microbiome. The plasma is accepted as having its own microbiome that can be impacted by disease.^16^ Any change in the number and composition of the microbiome impacts tissue function and contributes to disease progression. Next, we identify the dominant bacterial genera in the fecal, plasma, globes, and retina of Akita mice and compared them with WT mice. Previously, we showed in feces of Akita mice reduced microbial populations of *Akkermansia, Bifidobacterium, Lactobacillus,* and increased *Bacteroides* compared to WT mice.^11^ In agreement with what we observed in the feces, *Akkermansia, Bacteroides, Bifidobacterium, Clostridium, Corynebacterium, Faecalibacterium, Lactobacillus, Propionibacterium, Pseudomonas*, and *Staphylococcus* are the top 10 genera identified in plasma and globes. Reduced abundance of *Akkermansia, Bacteroides, Bifidobacterium, Clostridium, Faecalibacterium, Lactobacillus,* and *Staphylococcus*, and increased abundance of *Corynebacterium,* and *Propionibacterium* were observed in plasma samples (Fig. 4A; left panel) of Akita mice compared to WT mice. In the globes of Akita mice (Fig. 4A; right panel) lower levels of *Akkermansia, Corynebacterium, Faecalibacterium,* and *Staphylococcus* were observed than in the WT mice whereas a higher abundance of *Bacteroides, Bifidobacterium, Clostridium, Lactobacillus, Propionibacterium*, and *Pseudomonas* was observed. In the retina of Akita mice (Fig.4B), levels of *Lactobacillus,* *Staphylococcus,* and *Enterococcus* and *Bacillus* were higher than the WT mice whereas lower levels of *Corynebacterium* and *Pseudomonas* were noted.

### Enrichment of Functional Pathways in Globes and Retina of Akita Mice

**Figure 5.**
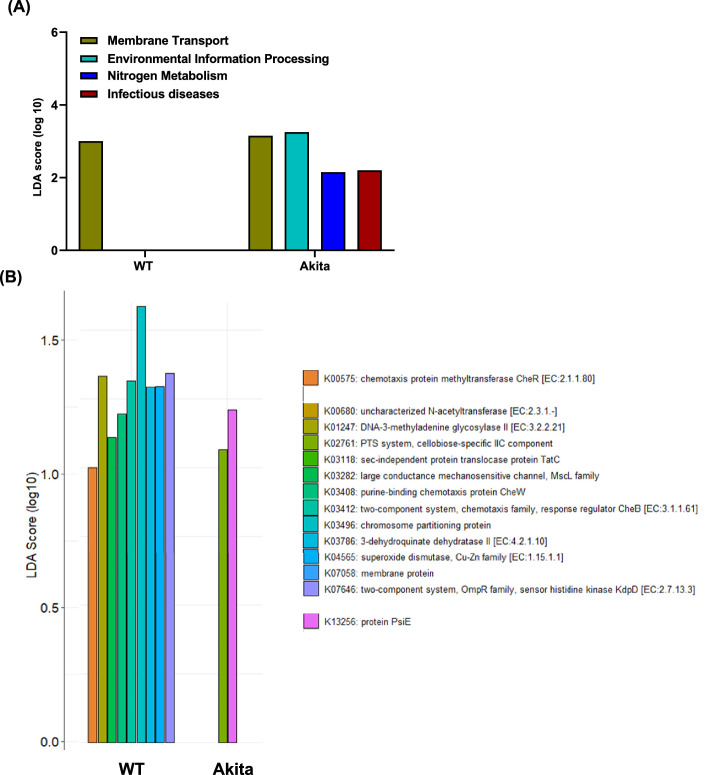
Identification of functional pathways in the globes and retina of Akita mice. The functional pathways influenced by microbial imbalance in the globes of diabetic mice **(A)**. Akita mice appeared to have three defined functional expression profiles: Environmental information processing, nitrogen metabolism, and infectious diseases. In the retina, two different pathways, PST system and protein PsiE, were noted in Akita mice **(B)**.

Enriched functional gene pathways expressed in globes of Akita and WT mice are shown as LefSe plots (Fig. 5A). Three pathways were identified to be different in the globes of Akita mice versus WT mice. The KEGG BRITE databases showed environmental information processing, nitrogen metabolism, and infectious diseases. These pathways describe key signaling cascades including MAPK kinase, ErbB, Ras, Wnt, Hedgehog, transforming growth factor-beta, vascular endothelial–derived growth factor (VEGF), Apelin, JAK-STAT, NF-kappa B, tumor necrosis factor, HIF-1, PI3K/Akt, AMPK, mTOR signaling, energy metabolism, and virus and bacterial infections.

In the retina samples obtained from Akita mice perfusion with saline solution to eliminate the contribution of the plasma microbiome, the PTS system cellobiose-specific IIC component (K02761) was identified (Fig. 5B). This KEGG pathway is associated with energy metabolism. The K13256 protein PsiE represents *Escherichia coli*, which is a strain known to be increased in response to starvation particularly in low phosphate.

### Increased Levels of PGN and TLR2 in the Retina of Akita Mice

**Figure 6.**
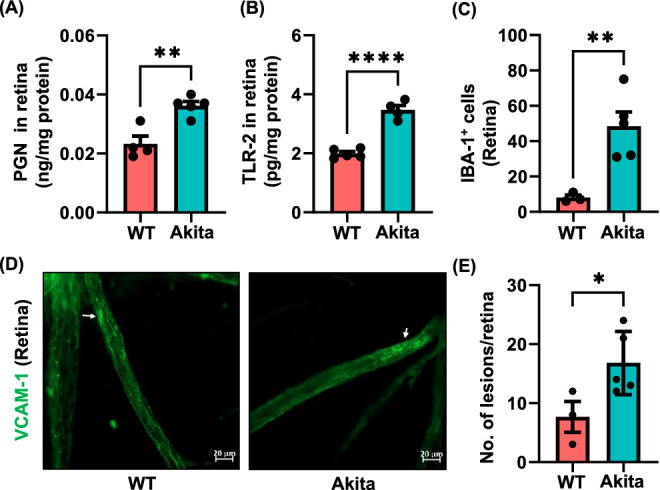
Levels of PGN, TLR-2, and inflammation in the retina of Akita mice. The levels of PGN **(A)** and TLR-2 **(B)** were significantly higher in the retina of Akita mice compared with the WT cohort. The number of activated Iba-1^+^ cells in the retina of both cohorts were quantitated **(C)**. The expression of VCAM-1 was immunohistochemically detected in retina **(D)** and VCAM-1 fluorescence was quantified **(E)**. Data are presented as mean ± SEM. Each *circle* represents n number in the cohorts. Each *dot* represents individual sample.

The eye, once thought to be a sterile and immune privileged site, has not only a surface microbiome but also contains microbes in the retina. We observed a higher abundance of gram-positive bacteria (Fig. 4A). Therefore we next measured the levels of PGN in the retina and found significantly higher in the Akita mice (0.036 ± 0.001) compared to the WT (0.023 ± 0.002; *P* < 0.002) (Fig. 6A). These observations suggest that gut microbes, translocate from the intestine into bloodstream and may enter the eyes through systemic circulation.

TLR-2, a receptor of PGN, plays an important role in the immune system in response to numerous pathogens.^24^ On recognition of microbial products including PGN, TLR-2 becomes activated and initiates a cascade of molecular signaling promoting inflammation and oxidative stress.^11^ We found higher levels of TLR-2 in the retina of Akita (3.47 ± 0.15) compared to WT mice (1.99 ± 0.07; *P* < 0.0001; Fig. 6B).

### Assessment of Retinal Inflammation in Akita Mice

Microglial cells are the tissue's resident macrophages and a major source of pro-inflammatory cytokines in the retina. As seen in Figure 6C, the number of activated Iba-1^+^ microglial cells (retracted processes and cell body) were significantly higher versus in the retina of Akita mice (48.4 ± 8.1) compared to WT mice (8.0 ± 1.52; *P* < 0.009).

Vascular cell adhesion molecule 1 (VCAM-1) is an inducible glycoprotein that is predominantly expressed in endothelial cells. VCAM-1 expression is increased by pro-inflammatory cytokines, high glucose and TLR agonists. As seen in Figure 6D, retinal endothelial cells of Akita mice demonstrated increased expression of VCAM-1 compared to WT mice. Semi quantification of VCAM-1 immunofluorescence was higher in the retina of Akita mice (5.598 ± 0.65) compared to WT (2.98 ± 0.74; *P* < 0.04) mice (Fig. 6E).

### Impaired Retinal Function and Increased Acellular Capillaries in the Akita Cohort

**Figure 7.**
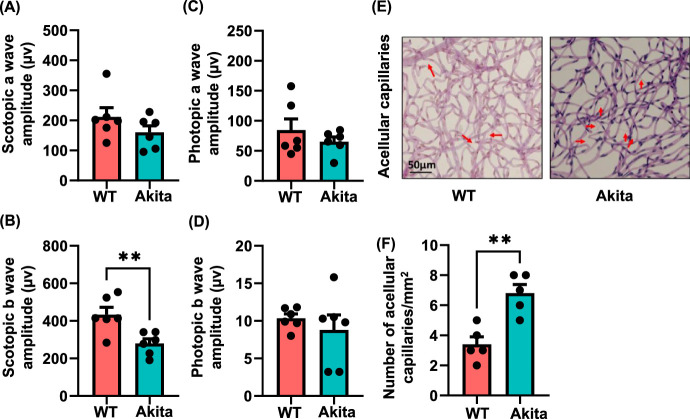
Assessment of visual function and acellular capillary in Akita and WT littermates. Visual function in Akita mice was assessed by ERG and was compared with WT mice. The amplitude of a-waves and b-waves was measured in both scotopic **(A-B)** and photopic **(C-D)** conditions after nine months of diabetes. Representative images of acellular capillaries in Akita and WT mice. *Red arrows* indicate acellular capillaries in the retinas **(E)**. Enumeration of acellular capillaries is summarized and presented as mean number of acellular capillaries/mm^3^ ± SEM. Each *circle* represents n number per group **(F)**. Each *dot* represents individual sample.

At nine months of diabetes, the Akita mice demonstrated no reduction of scotopic a wave (160.2 ± 21.77) compared to age-matched WT mice (211.3 ± 31.38; *P* < ns); however, the scotopic b wave (279.2 ± 24.84) was decreased compared to age matched WT mice (433.1 ± 39.3; *P* < 0.0007) (Figs. 7A, 7B). No significant reduction in photopic a wave (65.12 ± 7.94 vs 84.67 ± 18.65; *P* < ns) or photopic b wave (8.8 ± 1.98) was observed in Akita mice (Figs. 7C, 7D) compared to WT mice (10.33 ± 0.58; *P* < ns).

As shown in Figure 7E, significantly higher number of acellular capillaries was observed in Akita mice compared to WT mice. The number of acellular capillaries was twofold (6.8 ± 0.58) in Akita mice compared to their wildtype littermates (3.4 ± 0.51; *P* < 0.002) (Fig. 7F).

## Discussion

Recently, the contribution of the gut microbiome^25^ to the generation of a plasma microbiome and its role in increasing metainflammation in the systemic circulation have become appreciated. Here, we sought to determine whether the diabetic gut microbiome might impact gut barrier integrity, allow microbes and microbial products to enter the circulation resulting in changes in the plasma microbiome. In turn, serving to activate the retinal endothelium and contribute to DR pathogenesis. We found that the diabetic globe and retina retain bacteria that are likely derived from the gut due to loss of both the gut barrier in diabetes and the retinal barrier integrity. These microbes can contribute to DR by further stimulating inflammation in the intraocular environment. The interior of the eye has historically been understood as “immune-privileged” suggesting that the tissue is free of bacteria and other immune stimulatory molecules. The features of the eye that allow this immune privilege include the lack of efferent lymphatics and the tight junctions of the blood-retina barrier (BRB) that regulate the restriction of pathogens and maintains ionic gradients.^26^ Other means of immune privilege features are through cell-bound immunosuppressive growth factors such as TGF-β and α-MSH.^27^

However, the BRB serves as a dynamic barricade for the maintenance of “immune-privilege” of the ocular environment when the environment is in a non-diseased homeostatic state. In the diseased environment of DR, increased amounts of inflammatory leukocytes adhere to the endothelial cells of the retina via upregulated VCAM-1 and in turn release inflammatory cytokines such as TNF-a and IL-1B that contribute to interruptions of tight junctions and a transient disruption of BRB during retinal inflammation.^26^^,^^28^^–^^30^ As presented in this study, the leakage of gut microbes and microbial peptides contribute to an intraocular inflammatory environment and BRB breakdown. Circulating gut-derived peptides such as PGN enter the systemic circulation and activate TLR2 on endothelial cells contributing to retinal barrier leakage.^11^^,^^31^ Thus the combination of the underlying systemic inflammatory state and the resulting leakage of microbial products into the circulation seen in diabetes contribute to the BRB dysfunction driving the loss of “immune-privilege” state of the ocular system.^32^^,^^33^ In this study, we were cognizant of the role of the ocular surface microbiome and removed the contribution of these microbes from the globe microbiome (Figs. 1C, 2C, 3B, 4A, and 5A). We isolated the retina under sterile conditions from the Akita mice after perfusing the mice with sterile saline to eliminate the plasma microbiome.

Although the precise mechanism of how the change in the diabetic gut microbiome effects DR is still unknown, our study shows that the microbiome composition in the gut and plasma can potentially affect the inside of the ocular globe and retina. These microbes that find their way to the eye, will even transiently, may contribute to the pathological changes associated with DR by activation of TLR2/4 and subsequent downstream signaling.

In the intestine, the mucus layer is vital for maintaining mucosal barrier integrity.^34^ Impaired intestinal barrier integrity leads to microbiota imbalance, facilitates bacterial and endotoxin translocation,^35^ and promotes systemic inflammatory response and multiple organ dysfunction.^36^^,^^37^ Despite a role in mucin degradation, *Akkermansia* also simulates mucin synthesis through an autocatalytic process^38^^–^^40^ thus they are considered a beneficial bacterium in maintaining the gut barrier^41^ and are lost in Akita mice. The gut microbiota produces active metabolites including lipopolysaccharides (LPS). Species of *Bacteroidetes*, gram-negative bacteria involved in LPS production,^42^ have deleterious effect on the gut barrier^43^ and are increased in Akita mice (Fig. 3A). *Bacteroidetes* is a phylum of well-known gram-negative anaerobe derived from the gut and involved in carbohydrate metabolism. Although this species has a beneficial effect on intestinal metabolism, once this bacterium leaves the gut, it can quickly become destructive to tissues.^44^ Its unique bacterial polysaccharide capsule, made of LPS, allows it to induce abscess formation.^45^ Proinflammatory cytokine release and leukocyte adhesions are known to be early mechanisms of DR.^46^ LPS has been shown to enhance the hypoxic response created in DR by increasing the IL-6 and IL-8 expression in human retinal pigment epithelium cells,^47^ thus indicating that the increased presence of this unique bacterial capsule within the eye could contribute to retina inflammation.

*Enterococci* are gram-positive bacteria that are common in the gastrointestinal tract of almost all land animals.^48^ The genera enterococcus were increased in abundance in the Akita retina compared to the WT retina (Fig. 4B). They play a specific role in the gut microbiome by aiding in nutrient digestion and development of gastrointestinal mucus barrier immunity.^49^ However, as the microbiome in a diseased inflammatory state leads to dysbiosis, *Enterococci* tend to overpopulate and disrupt the symbiotic relationship previously described, leading to gut-barrier disruption and leakage of microbial products into systemic circulation. These processes contribute to the induction of a systemic inflammatory state and autoimmune disease.^50^ Furthermore, studies in which Enterococci were overpopulated in the gut via pharmacologic mechanisms resulted in animals predisposed to developing full blown autoimmune disease with Enterococci proliferated in other internal organs outside the site of transplantation.^50^ Another study investigated the effects of gut-leakage of *Enterococci* by directly inoculating the liver and other tissues with this microbe and found that the translocation of *Enterococci* resulted in autoimmune disease due to systemic inflammation and activation of TLRs.^51^ Through the breach in gut-barrier integrity and its impact on systemic inflammation through activation of TLRs, the presence of *Enterococci* in the diabetic retina has the potential to contribute to the inflammatory ocular environment observed in DR.

*Lactobacillus* is a well-studied gram-positive microbe that is important in the gut and plays a key role in carbohydrate metabolism. This bacterium is an active ingredient in several probiotics that have been shown to have beneficial effects on irritable bowel syndrome, cholesterol assimilation, atopic dermatitis, carbohydrate metabolism, and weight loss.^52^ Studies have shown that in the diabetic gut, *lactobacillus* is reduced in abundance, and with its reduction there can be dysregulation of carbohydrate metabolism contributing to elevated blood glucose. Previously, we showed a lower abundance of *lactobacillus* in the gut of Akita mice^11^ and in this study we also observed a reduction in *Lactobacillus* in the plasma of Akita mice (Fig. 4A). *Lactobacillus* has a protective effect on the gut-epithelium, suggesting that the loss of *lactobacillus* could promote leaking of microbes and microbial products. However, although *lactobacillus* has a beneficial symbiotic effect on carbohydrate metabolism in the gut environment, studies have shown that *Lactobacillus* has a pathobiont effect dependent upon the context of its location and proliferation.^50^ The variable effects that *Lactobacillus* has on the development disease are dependent on the conditions on which the microbes use.^53^ *Lactobacillus* is one of a group of bacteria that undergo homofermentative metabolism and generate lactic acid as their end product. Lactic acid can activate GPR81, a G-coupled cell-surface receptor^54^ localized on retinal ganglion cells and Muller cells.^55^ Although not tested in our study, bacterial lactic acid could potentially be a source of lactic acid that activates GPR81 downregulating cAMP impacting protein kinase A and cyclic nucleotide channels. Lactic acid can also modulate VEGF expression and produce numerous angiogenic factors including Norrin, which can contribute to vascular proliferative changes.^56^^,^^57^ Although we can only speculate, increased *Lactobacillus* in the intraocular globe and retina of Akita mice might contribute to the pathogenesis of DR by these previously addressed mechanisms.

Among several genera, *Bacillus* was found to be increased in abundance in the Akita retinas. *Bacillus* are well-studied spore-forming gram-positive bacteria and exists as either an aerobe or facultative anaerobe.^58^ They exist in low abundance in the gut-microbiome, but because of their vast range of secreted compounds, they can have major impact on the gut epithelial lining and barrier integrity,^59^ especially in an already predisposed diseased state. Of further interest, *Bacillus* is one of the main causes of endogenous endophthalmitis (EBE), a severe intraocular infection originating from the blood stream.^60^^,^^61^ Diabetes is the leading predisposing risk factor for the development of EBE,^62^ and the compromise in BRB in DR may be linked to this pathogenesis.^32^^,^^33^ *Bacillus* has been shown to compromise the BRB via the loss of both ZO-1 and occludin,^63^ as well as RPE cell destruction and contribution to retinal inflammation through production of proinflammatory cytokines IL-6 and IL-1β.^44^ Our findings of *Bacillus* abundance in the diabetic retina in concordance with *Bacillus’* potential to create barrier leakage and an intraocular inflammatory environment, indicate that increased presence of this microbe in the retina has the potential to further the development of DR.

The finding that the Akita globes and retinas exhibited more bacterial taxa coincides with the implication that BRB dysfunction occurs in diabetes and may contribute to DR.^64^ From characterization of these retinas, we found evidence of DR by both ERG and acellular capillaries (Figs. 6 and 7). Although the BRB has traditionally contributed to establishing the “immune privilege” of the ocular environment, its disruption in DR can lead to leakage of macromolecules such as gut microbial peptides^65^ and promote bacterial entry from the plasma into the retina. Recent evidence shows that the outer BRB contributes to DR progression as well and can be seen through the dysregulation of transport leakage of macromolecules.^66^ This dysregulation of both the outer and inner BRB suggests that compromise of these barriers allows translocation of plasma born microbes into the retina and can contribute to development of DR. Inflammatory changes in the retina can be seen through various mechanisms such as the increase in acellular capillaries in the retina, an increase of VCAM, and the increase in Iba-1.^67^^,^^68^

In summary, our study brings attention to the existence of a plasma microbiome in both WT and Akita mice. In diabetes, this plasma microbiome can result in the presence of potentially pathogenic microbes in the retina contributing to DR pathogenesis. By the characteristic breach in BRB associated with DR the ensuing increase in retinal bacterial products may activate signaling pathways such as TLRs and GPR81. This activation can further stimulate the local release of cytokines and VEGF supporting a role of the gut-retina axis in DR pathogenesis.
